# Spatiotemporal and metabolic heterogeneity of tumor-associated macrophages in glioblastoma: from single-cell insights to therapeutic targeting

**DOI:** 10.3389/fcell.2026.1774215

**Published:** 2026-03-26

**Authors:** Jun Lu, Siwen Chu, Shisong Wang, Siqi Wang, Zhongxue Yu, Zihao Yan, Guangyang Ji, Hongxu Zhou, Jun Wang, Chen Zhu

**Affiliations:** 1 Department of Neurosurgery, The First Hospital of China Medical University, Shenyang, Liaoning, China; 2 Department of Ultrasound, The First Hospital of China Medical University, Shenyang, Liaoning, China; 3 Department of Radiation Oncology, The First Hospital of China Medical University, Shenyang, Liaoning, China; 4 Department of Cardiovascular Ultrasound, The First Hospital of China Medical University, Shenyang, Liaoning, China; 5 Department of Neurosurgery, Liaoning Cancer Hospital and Institute, Cancer Hospital of China Medical University, Cancer Hospital of Dalian University of Technology, Shenyang, China; 6 Department of Anesthesiology, The First Hospital of China Medical University, Shenyang, Liaoning, China

**Keywords:** combination therapy, glioblastoma, heterogeneity, immune microenvironment, tumor-associated macrophages

## Abstract

The immunosuppressive and therapy-resistant nature of glioblastoma (GBM) is fundamentally driven by the profound spatiotemporal and metabolic heterogeneity of tumor-associated macrophages (TAMs). This review proposes a spatiotemporal-metabolic axis as an integrative framework to decipher the functional plasticity of TAMs and its therapeutic implications. Drawing on the latest single-cell and spatial multi-omics data, we first delineate the lineage competition landscape of TAMs. Within this landscape, brain-resident microglia, border-associated macrophages (BAMs), and peripherally recruited bone marrow-derived macrophages (BMDMs) engage in dynamic interplay during tumor evolution, culminating in a shifted ecosystem dominated by BMDMs at recurrence. These subsets are not randomly distributed but are spatially organized through niche-instructive signals—such as hypoxia, perivascular cues, and tumor-derived metabolites—leading to context-dependent enrichment: immunosuppressive TAMs accumulate in the tumor core, BAMs localize to perivascular zones and express pro-angiogenic factors, while hypoxic necrotic regions are populated by metabolically reprogrammed HMOX1^+^ TAMs. Metabolically, TAMs engage in symbiotic nutrient exchange with glioma cells via enhanced glycolysis, amino acid catabolism, and lipid accumulation, collectively reinforcing an immunosuppressive microenvironment. Building on this multidimensional understanding, we highlight emerging therapeutic strategies that move beyond broad depletion: metabolic-epigenetic interference (e.g., targeting lactate-driven histone lactylation), phagocytosis checkpoint blockade (e.g., CD47-SIRPα axis), and niche-precise targeting of hypoxic or perivascular TAM subsets. This review provides an integrative roadmap for developing next-generation immunotherapies that leverage the spatiotemporal and metabolic logic of TAMs to reprogram the GBM microenvironment.

## 1 Introduction

Glioblastoma (GBM), the most aggressive primary brain tumor, exhibits profound heterogeneity across genetic, cellular, metabolic, and microenvironmental dimensions, which collectively drive its aggressive pathogenesis (Marusyk et al., 2020; Nicholson and Fine, 2021; Mathur et al., 2024). Despite maximal therapy—including surgical resection, radiotherapy, and temozolomide—median survival remains ∼15 months due to inevitable recurrence and therapeutic resistance (Lapointe et al., 2018; Stupp et al., 2005). Central to this challenge is the immunosuppressive tumor microenvironment (TME), where tumor-associated macrophages (TAMs) emerge as the dominant immune cell population. TAMs orchestrate immunosuppression and tumor progression through extensive crosstalk with neoplastic and stromal cells (Hoogstrate et al., 2023; Friebel et al., 2020; Cui et al., 2020).

While historically categorized by the M1/M2 dichotomy, this framework is now recognized as overly simplistic and incapable of capturing the functional and spatial diversity of TAMs, as revealed by single-cell and spatial omics technologies (Strizova et al., 2023). These advances have instead identified distinct TAM subsets—such as hypoxic, proliferative, and interferon-responsive states—defined by their ontogeny (BMDMs vs. microglia), metabolic programs, and niche-specific functions (Dusoswa et al., 2020; Chuang et al., 2013; Zhao et al., 2021; Wang W. et al., 2024; Ravi et al., 2022). Critically, the spatial architecture of TAMs is not one of rigid compartmentalization, but is best understood as a model of preferential enrichment within specific niches, shaped by local environmental cues, such as hypoxia, vascular signals, and direct interactions between tumor cells.

To systematically deconstruct the complexity of TAMs in GBM, this review is structured around three principal axes of heterogeneity: functional diversity (encompassing molecular and metabolic programs), context-dependent spatial distribution across and beyond classical anatomical niches, and temporal evolution from primary to recurrent disease. We synthesize recent insights to move beyond a static anatomical map, examining instead the dynamic principles that govern TAM localization, function, and adaptation throughout tumor progression.

## 2 The derivation and composition of TAMs in GBM

TAMs in gliomas originate from brain-resident populations (microglia and BAMs) and BMDMs (Dusoswa et al., 2020). Higher TAM density is a well-established feature of advanced tumor grade and is correlated with immunosuppression and poor prognosis (Liu et al., 2022). This association remains significant in the context of recurrent GBM (Knudsen et al., 2024). Interestingly, upon recurrence, the GBM microenvironment undergoes a remodeling of its immune landscape. Single-cell studies reveal a decrease in the relative proportion of TAMs among CD45^+^ cells, concurrent with a marked shift in their composition characterized by a significant increase in the proportion of BMDMs relative to microglia (Pombo Antunes et al., 2021). This altered BMDM-enriched population continues to drive tumor progression and therapy resistance. Recent advances in specific markers (e.g., TMEM119 vs. CD14/CD16) and single-cell technologies now resolve the functional heterogeneity between these subsets, revealing distinct spatiotemporal distributions and niche adaptations (Friebel et al., 2020). Although other immune cells, such as MDSCs (Myeloid-Derived Suppressor Cells), may exhibit macrophage-like characteristics under hypoxic conditions, current evidence regarding their contribution to the TAM population in GBM remains limited (Tcyganov et al., 2018; Srivastava et al., 2019; Sun et al., 2024).

### 2.1 Bone marrow-derived macrophages

**FIGURE 1:**
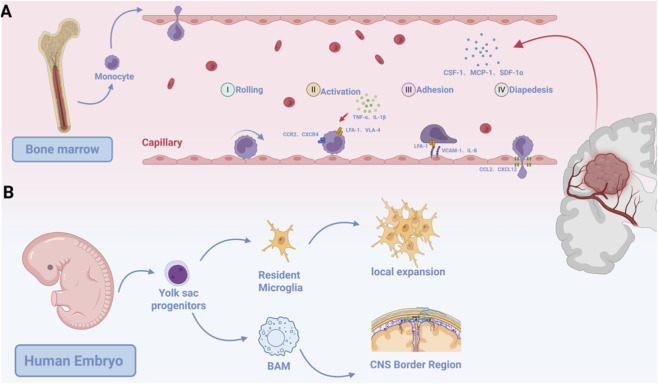
The derivation and composition of TAMs in GBM. **(A)** Originate from circulating monocytes and infiltrate the tumor via classical leukocyte recruitment mechanisms. **(B)** Yolk sac-derived microglia self-renew in the CNS. Similarly derived BAMs reside at CNS borders and are recruited into tumors.

In GBM, bone marrow-derived infiltrating leukocytes predominantly consist of BMDMs (Figure 1A). The characteristics and functions of BMDMs in GBMs may vary significantly depending on molecular subtypes, patient specific characteristics, and prognosis (Friebel et al., 2020). In both mouse models and human GBM, bone marrow-derived macrophages (BMDMs), which primarily originate from peripheral blood monocytes, are recruited to the tumor via the circulation during early tumorigenesis and constitute a major component of the TAM pool (Wang W. et al., 2024). Recent single-cell transcriptomic studies have solidified the molecular identity of BMDMs by identifying a core set of signature genes, including transcriptional markers (e.g., S100A8/A9, SPP1, TGFBI) and surface proteins (e.g., CD14, ITGA4/CD49D) (Pombo Antunes et al., 2021; Wang F. et al., 2024). This gene expression signature clearly separates them from resident microglia in bulk and single-cell datasets, independently of spatial context. The recruitment process follows the general paradigm of leukocyte trafficking, including rolling, adhesion, and transmigration. Tumor-derived factors (CSF-1, MCP-1, SDF-1α) and astrocyte-secreted CCL2 recruit circulating monocytes to perivascular zones (Zhang et al., 2023; Perelroizen et al., 2022). Subsequent BBB(Blood-brain barrier) disruption—mediated by VEGF/TNF-α-induced vascular permeability and MMP (Matrix Metalloproteinases)-dependent extracellular matrix degradation—enables monocyte transmigration into the tumor parenchyma (Pombo Antunes et al., 2021). Upon infiltration into the tumor parenchyma—a process critical for maintaining the TAM pool (Flores-Toro et al., 2020)—circulating monocytes undergo differentiation and functional adaptation. The precise temporal dynamics of this differentiation process are discussed in detail in Chapter 6 (Kirschenbaum et al., 2024) (Figure 1A).

### 2.2 Resident myeloid cells: Microglia and border-associated macrophages

Microglia are the predominant parenchymal resident macrophages of the central nervous system (CNS). They originate from yolk sac progenitors during embryonic development, with recent single-cell lineage tracing studies strongly supporting their derivation from erythroid-myeloid precursor cells (Wu et al., 2025; Khan et al., 2023) (Figure 1B). In the healthy brain, microglia populations are maintained through local self-renewal. Their identity is classically defined by a conserved transcriptional signature, including markers, such as P2RY12, TMEM119, and CX3CR1 (Khan et al., 2023, Utz et al., 2020).

However, the glioblastoma microenvironment profoundly challenges the use of these markers for strict lineage discrimination. A recent integrative multi-omics study of human glioma has revealed remarkable plasticity in myeloid cell identity, demonstrating that blood-derived monocytes can acquire the canonical microglial marker TMEM119 within 48 h of infiltrating the tumor (Miller et al., 2025). This finding critically informs the interpretation of TAM composition, indicating that marker expression alone may not be a definitive indicator of cellular origin, and that the TAM pool comprises cells with diverse ontogenies that can converge on similar molecular identities.

During early development, yolk sac progenitors also give rise to BAMs, which reside in specific CNS border regions, such as the meninges, choroid plexus, and perivascular spaces (Utz et al., 2020). Multiomic profiling has established a unique molecular identity for BAMs, characterized by high expression of LYVE1, FOLR2, and MRC1 (CD206) (Sankowski et al., 2024). This specific combination of markers, together with their characteristic lack of P2RY12 expression, provides a clearer toolkit to distinguish BAMs from both microglia(P2RY12^+^) and BMDMs, clarifying their independent contribution to the TAM pool. In the context of GBM, both resident microglia and BAMs can be recruited into the tumor mass and contribute to the TAM pool. The relative abundance and spatial distribution of these resident populations, as well as BMDMs, exhibit dynamic shifts across tumor regions and disease stages, which are explored in detail in Chapter 6 (Friebel et al., 2020, Pombo Antunes et al., 2021, Sankowski et al., 2024).

Beyond lineage markers, core macrophage identity is maintained by macrophage-specific genes including the MPEG family (Wu et al., 2025; Khan et al., 2023). MPEG1 (perforin-2), a pore-forming protein involved in intracellular bacterial killing, may also contribute to TAM-mediated phagocytosis in GBM (Miller et al., 2025). Emerging evidence suggests MPEG1 expression is dysregulated in immunosuppressive TAM subsets, potentially impairing phagocytic clearance of tumor cells (Wu et al., 2023). Restoring MPEG1 function could enhance tumor cell phagocytosis and synergize with CD47-SIRPα checkpoint blockade, though further research is needed to clarify its therapeutic potential.

## 3 The functional subtype and immune property of TAMs in GBM

The functional heterogeneity of TAMs forms a core layer of their diversity, transcending conventional conceptual frameworks (Strizova et al., 2023; Wu et al., 2020; Ransohoff, 2016). While these functional states are intrinsically linked to spatial localization and temporal context (as detailed in Sections 4, 6), this section categorizes TAMs based primarily on their specific pro-tumoral activities and histological associations. The classical binary model divides tumor-associated macrophages (TAMs) into M1 and M2 phenotypes with distinct genomic and functional profiles (Wang W. et al., 2024; Liu et al., 2024). Classically activated M1 macrophages exhibit a pro-inflammatory, anti-tumoral signature characterized by upregulation of IL-12, TNF-α, and NOS2(34). Alternatively activated M2 macrophages display an immunosuppressive, pro-tumoral signature marked by high expression of IL-10, TGF-β, ARG1, and MRC1 (CD206) (Sankowski et al., 2024). However, single-cell and spatial omics have revealed that TAMs in GBM rarely adopt these extreme states in isolation, instead existing along a continuum of activation states defined by unique molecular profiles and niche-specific functions rather than rigid binary categories (Wang W. et al., 2024; Liu et al., 2024). We will categorize TAM heterogeneity based on their specific functions (e.g., immunosuppressive, pro-angiogenic) or histologically-defined subgroups (e.g., hypoxia-associated, chemotherapy-associated).

For instance, studies in PTEN-deficient mouse GBM models have shown that macrophages with high LOX expression and microglia with high OLFML3 expression exhibit spatial exclusion, implying that they may play competing or complementary roles in tumor progression (Liu et al., 2024). In GBM (IDH-wildtype per WHO 2021), single-cell studies have resolved BMDMs into at least six functionally distinct subsets, among which are therapy-resistant TAMs characterized by adaptive changes following chemotherapeutic interventions (Wang W. et al., 2024; Sattiraju et al., 2023). Additionally, newly defined subsets include lipid-laden macrophages (LLMs), which are characterized by high CD36 expression and active lipid metabolism and promote tumor growth through lipid delivery (Kloosterman et al., 2024; Hale et al., 2014); perivascular TAMs, which are P2RY12-negative and highly express chemokines, such as CXCL12 and significantly promote angiogenesis (Friebel et al., 2020; Chen et al., 2017). Notably, this P2RY12^-^, pro-angiogenic phenotype is characteristic of BAMs recruited from CNS border structures into the tumor (Sankowski et al., 2024). Additionally, BAMs contribute to tumor progression by remodeling the extracellular matrix (ECM) through the expression of matrix metalloproteinases (MMPs) (Sankowski et al., 2024; Mundt et al., 2022).

**FIGURE 2:**
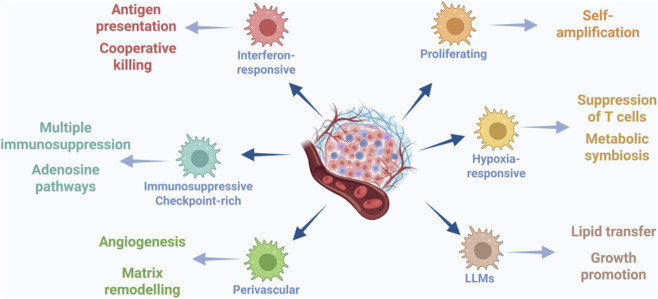
The functional subtype and immune property of TAMs in GBM. TAMs in glioblastoma exhibit functional and immunomodulatory heterogeneity. Distinct subsets with specialized transcriptional profiles collectively establish an immunosuppressive microenvironment and drive angiogenesis and therapy resistance.

These functionally distinct subsets exhibit specialized spatial distributions, as detailed in Section 6 (Figure 2).

The functional spectrum of TAMs in GBM may extend beyond purely pro-tumoral activities. It remains to be fully determined whether stable, anti-tumoral macrophage populations analogous to those described in other cancers exist and function within human GBM. For example, research in melanoma models has demonstrated that T cell-derived cytokines can re-educate TAMs toward a phenotype that synergizes with CD8^+^ T cells (Trotta et al., 2025). Critical unanswered questions are whether this potent anti-tumor mechanism can be operational in GBM, and how such putative anti-tumoral TAMs could be sustained or therapeutically induced against formidable immunosuppressive pressures. Exploring this extreme functional heterogeneity remains essential for developing novel TAM-targeting strategies (Figure 2). Supplementary Table S1 summarizes the experimentally defined TAM subsets/states in GBM, their key defining features, spatial localization, and primary functions, providing a clear overview of these newly identified subsets.

## 4 Metabolic heterogeneity of TAMs

The functional plasticity of TAMs is underpinned by profound metabolic heterogeneity. Within the GBM microenvironment, metabolic reprogramming across glucose, amino acid, and lipid pathways directly enables TAMs’ immunosuppressive functions and supports tumor progression. Zman-seq analysis has identified epigenetic regulators, such as H3K27ac and ATAC-seq peaks, that are enriched in glycolytic and lipid-metabolism genes within specific TAM subsets, suggesting that metabolic reprogramming in TAMs is underpinned by accessible chromatin states that may be therapeutically targetable (Kirschenbaum et al., 2024).

### 4.1 Glucose metabolism

**FIGURE 3:**
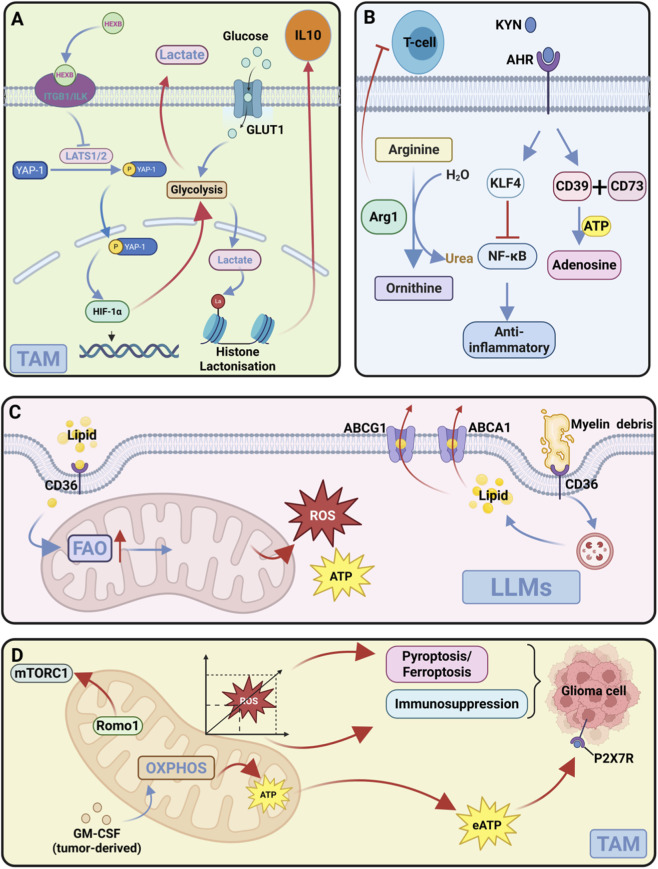
Metabolic heterogeneity of TAMs. **(A)** Glycolysis: Enhanced glucose uptake and lactate flux in TAMs promote histone lactylation and IL-10 expression. **(B)** Amino Acid Metabolism: Arg1-mediated arginine depletion inhibits T cells. Tumor-derived kynurenine activates AHR signaling, driving adenosine production via CD39. **(C)** Lipid Metabolism: Upregulated CD36 promotes lipid accumulation. Lipid-laden macrophages (LLMs) transfer lipids to tumor cells to support growth. **(D)** Mitochondrial Metabolism: GM-CSF induces OXPHOS/eATP activating P2X7R. Romo1-mTORC1 drives M2 polarization; ROS: moderate sustains immunosuppression, excessive triggers tumor death.

TAMs in GBM undergo significant alterations in glucose metabolism, distinguishing them from macrophages in normal tissues. Unlike the polarised macrophages observed in physiological conditions, Many GBM-associated TAM subsets, particularly those exhibiting immunosuppressive functions, predominantly adopt a glycolytic phenotype characterized by enhanced glucose uptake and lactate production. This metabolic reprogramming in immunosuppressive TAMs is mediated through multiple interconnected pathways. For instance, the hexosaminidase B (HEXB) protein activates glycolysis in both tumor cells and TAMs via the ITGB1/ILK/YAP1 signaling axis, creating a feed-forward loop that sustains their immunosuppressive phenotype (De Leo et al., 2024; Zhu et al., 2024). Simultaneously, the PERK-ATF4 pathway upregulates the glucose transporter GLUT1 in TAMs, thereby increasing glucose influx and subsequent lactate generation (De Leo et al., 2024). The resulting acidic microenvironment not only supports tumor cell survival but also induces histone lactylation in TAMs, epigenetically reinforcing their immunosuppressive properties through IL-10 upregulation (De Leo et al., 2024). Furthermore, IL-6 derived from immunosuppressive TAM subsets (commonly characterized as immunosuppressive functions) stimulates phosphoglycerate kinase 1 (PGK1) autophosphorylation in GBM cells, amplifying tumor glycolysis while creating a metabolic symbiosis (Figure 3A) between TAMs and malignant cells (Zhang et al., 2018).

### 4.2 Amino acid metabolism

The metabolic reprogramming of TAMs extends to various amino acid utilization pathways that collectively shape the immunosuppressive TME. Arginine metabolism represents a key regulatory node in GBM. Immunosuppressive TAMs express high levels of arginase 1 (Arg1), which depletes L-arginine to inhibit T cell function (Kirschenbaum et al., 2024; Ispirjan et al., 2024). This arginine depletion by TAMs is particularly significant in the GBM context, as a subset of tumor cells is auxotrophic for arginine due to low expression of ASS1, making the TME highly sensitive to arginine availability (Hajji et al., 2022). The synergistic arginine depletion by both TAMs and tumor cells creates a potent immunosuppressive barrier. Glutamine metabolism is profoundly rewired in GBM. Tumor cells exhibit “glutamine addiction,” avidly consuming it to support their rapid growth (Sponagel et al., 2022). Insights from non-GBM tumor models suggest that TAMs can upregulate glutamine synthase (GS) to maintain their polarization under nutrient stress (Palmieri et al., 2017). Supporting the relevance of this metabolic interplay in GBM, an *in vitro* model demonstrated that radiation therapy induces GS upregulation in GBM cells, and the resulting glutamine promotes the acquisition of an immunosuppressive phenotype in TAMs (Lu et al., 2025). This metabolic symbiosis formed within the GBM tumor microenvironment underscores the value of combined targeting. Branched-chain amino acid (BCAA) metabolism is notably altered in hypoxic regions, where GBM cells upregulate LAT1 transporters and BCAT1 enzymes to utilize BCAAs, with the resulting ketoacids potentially impairing macrophage phagocytic capacity (Zhang et al., 2021; Silva et al., 2017). Perhaps most significantly, the tryptophan-kynurenine-AHR axis creates a potent immunosuppressive circuit: tumor-derived kynurenine activates the aryl hydrocarbon receptor (AHR) in TAMs, inducing CCR2-mediated migration, M2 gene expression through KLF4, and CD39-dependent adenosine production (Figure 3B) that suppresses cytotoxic T cell activity (Takenaka et al., 2019).

### 4.3 Lipid metabolism

Lipid metabolic rewiring in TAMs contributes substantially to GBM progression through both energy provision and intercellular signaling. The limited capacity of GBM cells for *de novo* cholesterol synthesis creates a microenvironment where TAMs become crucial lipid suppliers. Through the upregulation of the fatty acid translocase CD36, TAMs enhance their lipid uptake capacity while exhibiting impaired cholesterol efflux mechanisms, resulting in intracellular lipid accumulation (Kloosterman et al., 2024; Hale et al., 2014). This metabolic adaptation forces TAMs to rely on fatty acid oxidation as their primary energy source, a shift that correlates with poor patient outcomes (Kloosterman et al., 2024; Su et al., 2020). Single-cell analyses have identified a specialized LLMs subset that actively participates in lipid transfer to tumor cells. These LLMs express high levels of lipid metabolism genes and engage in the phagocytosis of cholesterol-rich myelin debris, subsequently processing and transferring these lipids to fuel tumor growth (Kloosterman et al., 2024) (Figure 3C). The metabolic relationship between TAMs and GBM cells constitutes a true metabolic symbiosis—a self-reinforcing cycle of bidirectional nutrient exchange wherein TAMs supply metabolites that fuel tumor growth while tumor-derived signals lock TAMs into immunosuppressive states. This interdependence intensifies spatially within hypoxic niches, where nutrient scarcity forces tighter metabolic coupling. The resulting ecosystem is both tumor-supportive and immune-inhibitory, revealing therapeutic opportunities to simultaneously impair tumor progression and reverse immunosuppression by disrupting these symbiotic nodes.

### 4.4 Mitochondria: central hubs integrating metabolism and redox signaling in TAMs

Beyond these nutrient-centric metabolic pathways, mitochondria serve as central hubs integrating metabolic reprogramming, redox balance, and functional polarization in TAMs, yet their role in GBM has remained underexplored until recently (Wu et al., 2024; Venit et al., 2023). As key integrators of cellular metabolism and fate, mitochondria are now recognized to exhibit context-dependent functional states in TAMs. Contrary to the conventional view that immunosuppressive macrophages rely primarily on glycolysis, recent single-cell analyses have uncovered that TAMs in the GBM tumor core display elevated oxidative phosphorylation (OXPHOS) activity compared to those in the tumor periphery (Venit et al., 2023). Tumor-secreted GM-CSF drives this mitochondrial activation, leading to elevated extracellular ATP (eATP) production by TAMs, which subsequently activates P2X7R on glioma cells to promote tumor proliferation and invasion (Wu et al., 2024), establishing a self-reinforcing cycle of metabolic symbiosis(Figure 3D).

Reactive oxygen species (ROS) generated by mitochondria play dichotomous roles in the GBM microenvironment. Mitochondrial ROS modulator 1 (Romo1) promotes immunosuppressive M2-like polarization of BMDMs through mTORC1 signaling, with high expression correlating with poor prognosis (Sun et al., 2020). Conversely, excessive ROS accumulation can trigger pyroptosis and ferroptosis in glioma cells (Guo et al., 2026). This dual nature—promoting immunosuppression at moderate levels while inducing tumor cell death at high levels—presents both opportunities for therapeutic intervention by pushing ROS levels beyond a critical threshold. Therapeutic avenues include targeting the eATP-P2X7R axis, combining Romo1 inhibition with anti-PD-1 immunotherapy, and employing ROS-generating nanoparticles to promote TAM repolarization (Wu et al., 2024; Sun et al., 2020; Wang et al., 2022).

## 5 TAMs Sustain Immunosuppressive TME via Cellular Crosstalk

### 5.1 Immunosuppressive network

Beyond their direct suppression of T cell function through established mechanisms, such as metabolic interference and checkpoint signaling, TAMs orchestrate a broader immunosuppressive network in GBM by actively shaping the fate of antitumor immunity. A pivotal axis of this network is the collaboration between immunosuppressive myeloid cells and regulatory T cells (Tregs). The foundational role of Tregs in this interaction is well established, ranging from early work showing that they promote TAM polarization (Wainwright et al., 2011) to recent studies demonstrating that their depletion triggers profound pro-inflammatory activation of the myeloid compartment (Galvez-Cancino et al., 2025). Critically, in GBM, TAMs act as key antigen-presenting cells that directly drive progenitor-exhausted T cells toward a terminal exhaustion state, thereby depleting the pool of T cells capable of responding to immunotherapy (Waibl Polania et al., 2025). This TAM-driven erosion of T cell competence is spatially organized, occurring within niches enriched for IL-10-producing immunosuppressive TAMs (Ravi et al., 2022), and is further reinforced by the concordant accumulation of these activated TAMs with Treg populations in regions, such as the tumor core (Quail and Joyce, 2017; Tamura et al., 2018). Together, these interactions create a multi-layered immunosuppressive circuit that potently suppresses and depletes effective antitumor T cell responses.

**FIGURE 4:**
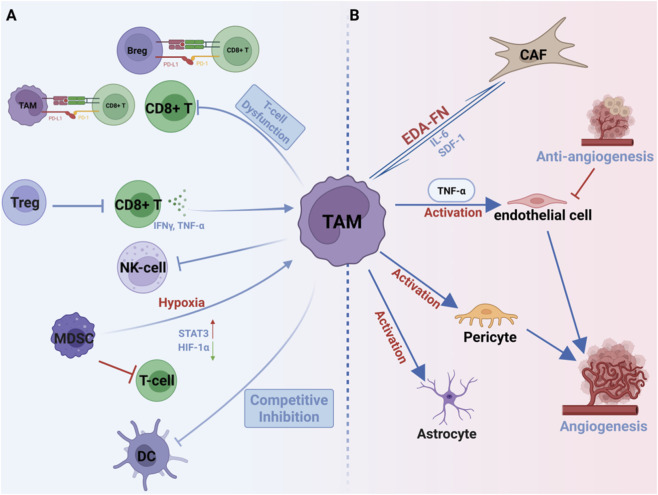
TAMs Sustain Immunosuppressive TME via Cellular Crosstalk. **(A)** TAMs inhibit T cells and NK cells, promote Treg expansion, impair dendritic cell function, and recruit other immunosuppressive myeloid cells. **(B)** TAMs drive angiogenesis, disrupt vascular barriers, and activate pericytes, astrocytes, and fibroblasts to reshape the stroma and support tumor progression.

The immunosuppressive landscape is further shaped by TAM interactions with other myeloid cells. Myeloid-derived suppressor cells(MDSCs) contribute significantly to this network, with monocytic MDSCs differentiating into TAMs under hypoxic conditions to establish immediate immunosuppression. In contrast, polymorphonuclear MDSCs maintain long-term immune tolerance (Kumar et al., 2016; Lin et al., 2024). Immunosuppressive TAMs also inhibit dendritic cell function through IL-10 secretion and direct competition via GPNMB expression, effectively blocking antigen presentation and T cell priming (Xiong et al., 2022; Sprooten et al., 2024) (Figure 4A). Single-cell transcriptomic studies utilizing techniques like Zman-seq have delineated the dynamic impairment of natural killer (NK) cells within the GBM TME, revealing that NK cells undergo a rapid functional transition within 24 h of tumor entry, shifting from a chemotactic (S1pr5-positive) and cytotoxic (Prf1/Gzma/Gzmb-positive) state to a dysfunctional one (Kirschenbaum et al., 2024). Critically, TAMs are positioned as primary drivers of this dysfunction through multiple direct mechanisms. TAMs are a major source of TGF-β within the GBM microenvironment, and this TAM-derived TGF-β directly suppresses NK cell cytotoxic function and limits the efficacy of adoptive NK cell therapies (Kirschenbaum et al., 2024; Rea et al., 2025). Beyond soluble factors, TAMs engage in direct contact-mediated suppression via surface expression of checkpoint ligands, including PD-L1 and CD47, which bind inhibitory receptors on NK cells and further impair their anti-tumor activity (Sankowski et al., 2024). This rapid TAM-driven functional erosion is a key factor underlying the limited efficacy of NK cell-based immunotherapies in GBM (Fares et al., 2023). B cells exhibit complex, context-dependent roles in this network, with tumor-associated Bregs maintained by MDSC-derived PD-L1+ extracellular vesicles contributing to local immunosuppression, while systemic B cell depletion paradoxically accelerates tumor progression (Lee-Chang et al., 2019; Pucci, 2019).

### 5.2 Stromal remodeling network

Beyond immune modulation, TAMs play a central role in reshaping the stromal microenvironment to support tumor progression and therapy resistance. Pathological angiogenesis is driven by TAM-secreted VEGF and TNF-α, which activate endothelial cells and upregulate adhesion molecules VCAM-1 and ICAM-1, promoting abnormal vascular remodeling (Wei et al., 2021). The limited efficacy of anti-angiogenic therapies can be attributed to compensatory expansion of pro-angiogenic TIE-2+ and CD206+ TAM subsets that emerge in response to treatment (Zhu et al., 2017a). Pericytes reinforce this process through mechanisms that promote pro-angiogenic TAM polarization. In other cancers, this involves IL-33/ST2 signaling (Yang et al., 2016), whereas in GBM, CECR1-mediated upregulation of periostin and VEGFA by TAMs reciprocally enhances pericyte function (Zhu et al., 2017b). These findings from different contexts converge on a similar model of pericyte-TAM collaboration that sustains vascular abnormalities (Figure 4B).

The blood-tumor barrier represents another critical stromal element shaped by TAM activity. Through the secretion of matrix metalloproteinases, TAMs increase vascular permeability; however, residual barrier integrity still significantly limits chemotherapeutic efficacy (Pombo Antunes et al., 2021; Abbott et al., 2006; Zhou et al., 2017). Astrocytes are co-opted into this immunosuppressive network through TAM- and tumor cell-derived cytokines, including IFN-γ and IL-6, which activate JAK-STAT signaling, transforming them into tumor-associated astrocytes that secrete additional immunosuppressive factors, such as TGF-β and G-CSF (Henrik Heiland et al., 2019). A compelling body of recent evidence now supports the discussion of the stromal network in GBM. First, the clinical relevance of CAF-like cells is established by findings that their abundance, although low, significantly correlates with higher tumor grade, poor patient outcome, and activation of extracellular matrix remodeling, with fibronectin (FN1) being a key functional mediator (Galbo et al., 2024). Furthermore, their cellular identity has been clarified, with FAP+/PDGFRβ+ tumor-associated pericytes identified as a major CAF-like stromal population (Li et al., 2020). Most critically, a specific LRRC15+ CAF subcluster has been mechanistically demonstrated to orchestrate the recruitment and pro-tumoral polarization of tumor-associated macrophages (TAMs) and confer resistance to anti-PD-1 immunotherapy via a reciprocal IL-8/TGF-β signaling loop (Luo et al., 2025). These GBM-specific studies validate a co-dependent and self-reinforcing circuit between CAF-like stromal cells and TAMs. While the specific molecular interactions in GBM are under active investigation, insights from other cancers suggest that analogous mechanisms, such as CAF-derived EDA-fibronectin engaging pattern recognition receptors (e.g., TLR4) on TAMs and TAM-derived factors (e.g., IL-6, SDF-1) reciprocally activating CAFs, may be operative (Mao et al., 2021; Bhowmick et al., 2004). The paradigm of functional synergy between the stromal and myeloid compartments in GBM is thus firmly supported by direct evidence (Galbo et al., 2024; Li et al., 2020; Luo et al., 2025; Jain et al., 2023).

## 6 Spatiotemporal heterogeneity and newly defined subsets of TAMs

Building on the established functional and metabolic diversity of TAMs, we now critically examine their spatiotemporal organization within GBM. The following sections first delineate the spatial heterogeneity of TAM subsets across major anatomical niches, and then detail the temporal dynamics governing their evolution from initial infiltration to recurrence. While a conventional view posits strict spatial compartmentalization of TAM subsets, emerging evidence supports a more nuanced model of context-dependent localization. Broad anatomical patterns do exist—such as the enrichment of BMDMs in the tumor core and resident microglia at the invasive margin—yet for many other TAM subsets, the link between spatial position and molecular identity is not absolute. Rather than fixed zoning, TAM distribution reflects a model of spatial preference shaped by niche-specific signals—such as hypoxia, damage-associated molecules, and direct interactions between tumor cells—that often transcend classical anatomical boundaries.

As previously outlined, the TAM pool in GBM comprises microglia, BAMs, and BMDMs, each exhibiting dynamic shifts in relative abundance across anatomical niches and disease stages (Friebel et al., 2020; Pombo Antunes et al., 2021; Sankowski et al., 2024). This spatial heterogeneity is further complemented by temporal evolution. Upon infiltration, circulating monocytes undergo rapid functional reprogramming, a process detailed by Zman-seq studies, which reveal that monocytes acquire immunosuppressive markers, such as Trem2, Il18bp, and Arg1, along a defined trajectory, peaking within 36–48 h via signaling pathways involving TGF-β1 and ANXA1 (Kirschenbaum et al., 2024). This time-resolved differentiation trajectory is conserved in both mouse models and human GBM, underscoring its clinical relevance and potential as a therapeutic window for targeting TAM polarization.

**FIGURE 5:**
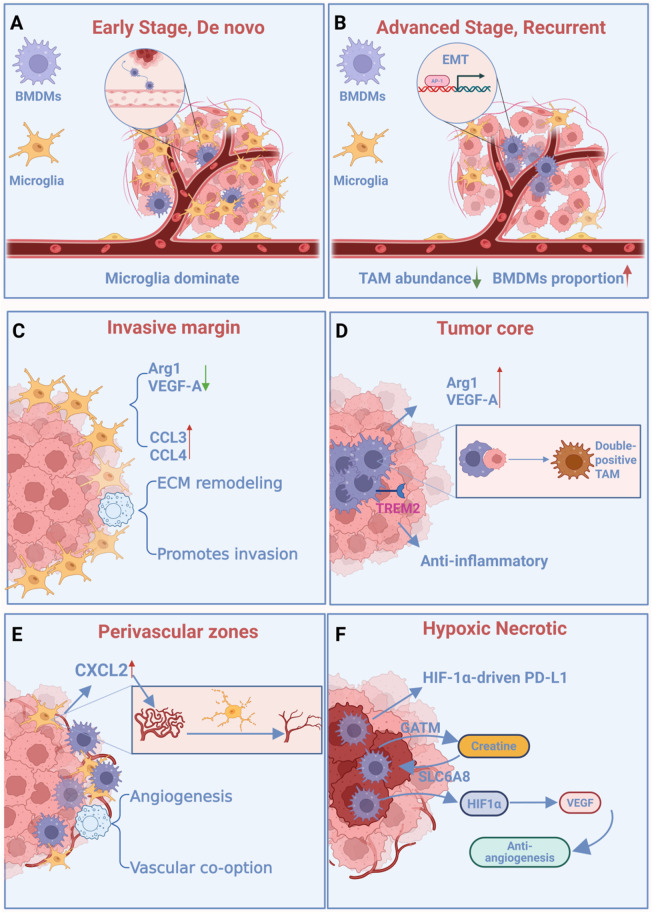
Spatiotemporal heterogeneity and newly defined subsets of TAMs. **(A)** Microglia dominate initial TAM populations; peripheral monocytes begin infiltrating the tumor. **(B)** TAM composition shifts toward BMDM predominance, correlating with enhanced therapy resistance. **(C)** Spatial gradient shaped by microglia-mediated recruitment and BAM-driven matrix remodeling; cerebral blood flow heterogeneity influences macrophage distribution. **(D)** Immunosuppressive TAMs dominate, marked by BMDM-associated markers and phagocytosis of glioma cells, promoting T cell dysfunction. **(E)** Co-localization of BMDMs and P2RY12^-^ pro-angiogenic macrophages; expression of checkpoint molecules and remodeling enzymes drives vascular and immune modulation. **(F)** Hypoxia-induced immunosuppressive polarization of TAMs; metabolic symbiosis via creatine synthesis (TAMs) and uptake (tumor cells) supports tumor growth.

Beyond spatial distribution, the TAM compartment exhibits profound temporal heterogeneity on two scales: the rapid functional programming of individual cells after infiltration, and the long-term remodeling of the entire TAM population during disease progression. Beyond these recruitment and differentiation timelines, the TAM ecosystem is also shaped by dynamic competitive interactions between resident and peripheral subsets. For example, in PTEN-deficient GBM, LOX inhibition can induce compensatory microglia infiltration, demonstrating the plasticity of the TAM compartment (Liu et al., 2024). Importantly, the TAM landscape undergoes dynamic remodeling during disease progression. In newly diagnosed GBM, resident microglia constitute the dominant TAM population. However, upon recurrence, the TAM compartment undergoes the ontogenetic shift described in Section 2, transitioning to a BMDM-dominated pool (Pombo Antunes et al., 2021) (Figures 5A,B).

Advances in single-cell transcriptomics and spatial multi-omics have enabled the systematic mapping of TAM subpopulations across spatial and temporal dimensions in GBM (Kirschenbaum et al., 2024, Li et al., 2022, Greenwald et al., 2024). However, the inherent disorganization of GBM and the technical limitations of spatial omics necessitate reliance on molecular markers (e.g., HMOX1, P2RY12) or histopathological features (e.g., pseudopalisading necrosis) to delineate tumor regions (Wang W. et al., 2024; Ravi et al., 2022). Conventionally, TAM distribution is categorized into four niches: tumor core, invasive margin, perivascular zones, and hypoxic necrotic regions (Wang W. et al., 2024; Bastola et al., 2020), with recent studies further identifying cerebral blood flow heterogeneity in peritumoral zones linked to TAM infiltration and recurrence (Wang F. et al., 2024). It is within this architectural context that we examine the spatial enrichment of TAM subsets, emphasizing that their localization is guided by a combination of structural, functional, and metabolic cues rather than rigid boundaries.

### 6.1 Tumor core and invasive margin

Spatially, the organization of TAM subsets across glioblastoma substructures extends beyond simple anatomical compartmentalization, reflecting instead a sophisticated interplay between cellular origin, functional specialization, and local microenvironmental cues. Integrated multi-region analyses utilizing intraoperative 3D navigation and single-cell omics have yielded two key findings. First, early tumor evolution involves peripheral regions where glioma cells co-opt neurodevelopmental programs. In contrast, late-stage tumor cores develop profoundly immunosuppressive microenvironments. These cores are dominated by TAMs expressing canonical immunosuppressive markers, such as CD206 and IL-10—often broadly categorized as M2-like—alongside tumor cells undergoing AP-1-driven mesenchymal transition (Mathur et al., 2024). The spatial dichotomy between core and periphery is conserved across model systems: murine GL261 gliomas show microglia enriched in the periphery and macrophages concentrated in the core (Ochocka et al., 2021), while human GBM transcriptomics confirm that core TAMs express BMDM-associated markers (CD14, S100A8/A9), and peritumoral TAMs exhibit microglial signatures (TMEM119, P2RY12, GPR34) (Darmanis et al., 2017). These peritumoral microglia are not static; they transition from a resting ramified state (P2RY12^+^, TMEM119^+^) in healthy brain to an activated amoeboid morphology with upregulated CCL3/CCL4 upon tumor encounter, and progressively lose homeostatic markers as they adopt dysfunctional TAM phenotypes in late-stage disease (Khan et al., 2023; Utz et al., 2020; Caponegro et al., 2021; Brandenburg et al., 2016). In recurrent GBM, this population is further diminished as BMDMs become dominant (Pombo Antunes et al., 2021). This consistent spatial segregation—BMDM-enriched cores and microglia-dominant peripheries—has been robustly validated across multiple single-cell and spatial omics studies (Wang W. et al., 2024; Sankowski et al., 2024; Greenwald et al., 2024). Notably, the phagocytosis of glioma cells by BMDMs reinforces their immunosuppressive phenotype, a mechanism that may solidify their dominance in the core during late disease stages (Wu et al., 2023). This fundamental niche specialization is further characterized by the exclusion of P2RY12^+^ microglia from the core, while P2RY12^-^ populations, including BAMs and BMDMs, occupy distinct compartments, such as the perivascular space and tumor core (Sankowski et al., 2024). While this represents a dominant anatomical pattern, it is best understood as a prevalent spatial preference rather than an immutable rule, as evidenced by the context-dependent distribution of other TAM subsets.

The immunosuppressive character of the tumor core is further emphasized by the elevated expression of Arg1 and VEGF-A compared to peritumoral brain zones, coupled with an increased CD4^+^/CD8^+^ T-cell ratio driven by Treg infiltration (Tamura et al., 2018) (Figures 5C,D). Beyond these broad patterns, functional studies reveal nuanced spatial logic among specialized TAM subsets. For instance, hypoxic TAMs (HMOX1^+^) described by Wang et al. are specifically adapted to the necrotic core (Wang W. et al., 2024), whereas IL-10–producing TAMs identified by Ravi et al. display a different distribution pattern, spatially coupling with tumor cell clusters exhibiting mesenchymal or astrocyte-like states, irrespective of whether these clusters reside in the core or invasive margin (Ravi et al., 2022). This indicates that the localization of IL-10^+^ TAMs is governed by functional interactions with specific tumor cell phenotypes rather than rigid anatomical boundaries.

At the invasive margin, a distinct functional gradient emerges, shaped by the interplay of tumor invasion and the neural microenvironment. The destruction of neuronal architecture and myelin integrity releases damage-associated molecules, such as phosphatidylserine and gangliosides, which activate TREM2 on myeloid cells and help establish a spatial polarization of TAM activity—typically more pro-inflammatory at the tumor–neuron interface and progressively immunosuppressive toward the core (Villa et al., 2024). The clinical significance of this peritumoral region is underscored by the fact that 90% of GBM recurrences originate within the peritumoral brain zone (PBZ) following resection (Lemée et al., 2015), making the functional characterization of TAMs in this niche prognostically critical. Recent studies have further refined our understanding of this area by identifying cerebral blood flow heterogeneity, which delineates higher (HBI) and lower (LBI) blood flow interfaces (Wang F. et al., 2024). Notably, macrophage infiltration is significantly elevated in high-flow regions (HBI: 8.02% vs. LBI: 4.90%), which are dominated by pro-angiogenic TAMs that colocalize with densely infiltrating tumor cells (Wang F. et al., 2024). This suggests that hemodynamic forces contribute to a specific immunosuppressive and pro-invasive microenvironment at the margin. Further adding to the functional complexity, a unique microglial subset at the tumor–stroma interface upregulates the pro-inflammatory chemokines CCL3 and CCL4, potentially facilitating the recruitment of peripheral monocytes and T cells (Caponegro et al., 2021; Yin et al., 2022). While the origin of this subset remains unclear, it may represent a transitional state that responds to—and actively shapes—the inflammatory landscape at the invasive front. Collectively, these findings illustrate that the invasive margin comprises a collection of dynamic, fluid-associated, and inflammation-modulated micro-niches, which collectively impose unique functional states on TAMs.

The spatial segregation of TAM subsets is not a static anatomical feature but is dynamically maintained through active molecular cross-talk. A key illustration of this plasticity comes from PTEN-deficient GBM models, where pharmacological inhibition of LOX in tumor cells triggers the NF-κB–PATZ1 signaling axis, leading to the upregulation of OLFML3 and a consequent compensatory infiltration of microglia (Liu et al., 2024). Among these, border-associated macrophages (BAMs), which are spatially enriched at the invasive margin, represent a critical component whose pro-tumorigenic functions are explored in the following section. Collectively, these insights affirm that the spatial architecture of TAMs is a product of continuous regulatory interplay, driven by structural, functional, and metabolic cues, which in turn reveal actionable targets for therapies aimed at both the immunosuppressive core and the invasive periphery.

### 6.2 Perivascular zones and hypoxic necrotic regions

The perivascular and hypoxic necrotic regions represent two critical spatial niches that exemplify how local microenvironmental signals dictate TAM identity and function. In the perivascular niche, a complex interplay of cell types and signals fosters a pro-angiogenic and immunosuppressive milieu. While BMDMs preferentially colonize CD31^+^ vascular areas (Friebel et al., 2020), this niche is co-inhabited by a distinct P2RY12^−^, pro-angiogenic macrophage population, phenotypically aligned with BAMs (Sankowski et al., 2024; Mundt et al., 2022). Upon recruitment into the tumor microenvironment, BAMs undergo significant metabolic reprogramming to acquire a potent pro-tumorigenic phenotype. This reprogramming enables them to coordinately upregulate immune checkpoint molecules (e.g., PD-L1, CD47) to suppress T cells, express matrix-remodeling enzymes (e.g., MMP14) to facilitate invasion, and secrete pro-angiogenic factors (e.g., VEGF) (Sankowski et al., 2024; Mundt et al., 2022). Single-cell analyses reveal that these infiltrating macrophages upregulate a suite of migration and remodeling genes (e.g., MMP9, TGFBI, CXCL12) compared to resident microglia (Chen et al., 2017; Quail and Joyce, 2017). Their polarization is further reinforced by CAFs present in microvascular regions (Jain et al., 2023). Notably, the contribution to angiogenesis is not exclusive to peripherally-derived macrophages; resident microglia themselves are implicated as pivotal drivers of vascular abnormalization via the CXCL2-CXCR2 axis, with their selective depletion significantly reducing microvascular density (Brandenburg et al., 2016). Paradoxically, anti-angiogenic therapies disrupt this equilibrium, exacerbating hypoxia and thereby activating HIF-1α, which in turn expands the very pro-angiogenic TAM populations that fuel therapeutic resistance (Zhu et al., 2017a) (Figure 5E).

This therapy-induced hypoxia intensifies the pre-existing harsh conditions of necrotic regions, which are architectural hallmarks of advanced GBM driven by thrombosis and oxygen diffusion limits (Rong et al., 2005). Spatial omics confirm that hypoxia is a master regulator of the tumor’s layered structure (Greenwald et al., 2024). Within these expanding necrotic areas, peripherally-derived TAMs, initially perivascular, infiltrate and become the dominant immune population as pseudopalisading structures mature—a hallmark of high-grade and recurrent disease (Pombo Antunes et al., 2021; Sattiraju et al., 2023). Hypoxia acts as a powerful instructing signal, recruiting TAMs and driving their immunosuppressive polarization largely through the HIF-1α pathway. HIF-1α directly upregulates PD-L1 on TAMs (Ding et al., 2021) and induces the expression of legumain (LGMN), a protease that further reinforces the immunosuppressive phenotype (Pang et al., 2023). Beyond suppressing immunity, these hypoxic TAMs (HMOX1^+^) overexpress hypoxia-response genes like ADM, BNIP3, and CSTB. Notably, ADM disrupts endothelial junctions, exacerbating vascular leakage and chemoresistance (Wang W. et al., 2024). At a more fundamental level, a profound metabolic symbiosis defines this niche: TAMs upregulate the creatine-synthesizing enzyme GATM, while GBM cells highly express the creatine transporter SLC6A8, allowing the tumor to directly utilize TAM-derived creatine to fuel its growth under nutrient stress (Rashidi et al., 2024) (Figure 5F).

In summary, the compartmentalization of TAMs across the perivascular and hypoxic niches is a clear demonstration of spatial enrichment guided by potent microenvironmental cues—from vascular and stromal signals to oxygen tension and metabolic demand. However, the presence of similar subsets, like pro-angiogenic TAMs, in both niches following anti-angiogenic therapy underscores that these distributions are not absolute. They reflect a context-dependent functional adaptation, where TAMs dynamically respond to the prevailing conditions to support tumor survival and resilience, thereby revealing a spectrum of therapeutic targets whose efficacy may be niche-dependent (Supplementary Table S1).

## 7 Targeting TAM heterogeneity: strategies and challenges

Therapeutic strategies must be evaluated by distinguishing preclinical from clinical evidence. The remarkable complexity and immunosuppressive nature of the human GBM microenvironment are not fully recapitulated in murine models, leading to frequent discrepancies between preclinical promise and clinical efficacy. Therefore, this section will explicitly categorize strategies based on their current stage of development—highlighting compelling preclinical findings while placing greater emphasis on the outcomes of clinical trials and the translational challenges they reveal. Therapeutic targeting of TAMs in GBM has been hampered by their remarkable spatiotemporal and metabolic adaptability, which enables rapid compensatory mechanisms in response to conventional therapies. While early strategies focused on depleting or repolarizing TAMs based on the M1/M2 dichotomy, emerging single-cell and spatial multi-omics data reveal a far more complex landscape of TAM subsets with context-dependent functions (Jin et al., 2025; Zhang and Jagannath, 2025). This complexity necessitates a refined approach that moves beyond broad phenotypic categorizations toward targeting specific functional states, metabolic dependencies, and niche-specific interactions (Ravi et al., 2022; Pombo Antunes et al., 2021).

### 7.1 Limitations of conventional TAM-targeting strategies

Preclinical studies in murine GBM models demonstrated that CSF-1R inhibition (e.g., with PLX3397) could effectively deplete pro-tumor TAMs and delay tumor growth. However, subsequent clinical trials in recurrent GBM patients (e.g., NCT01349036) revealed limited efficacy as monotherapy. This clinical failure has been attributed to compensatory mechanisms within the TME, such as the upregulation of the IGF-1/IGF-1R pathway in residual TAMs, which sustains immunosuppression and promotes tumor recurrence (Akkari et al., 2020). Similarly, anti-angiogenic agents (e.g., bevacizumab) exacerbate hypoxia, amplifying HIF-1α-driven TAM recruitment and polarization toward pro-angiogenic and immunosuppressive states (Zhu et al., 2017a). Even immune checkpoint inhibitors (ICIs) face resistance via upregulation of alternative immunosuppressive mechanisms, such as CD73-mediated adenosine production in hypoxic niches (Takenaka et al., 2019) or lipid-mediated phagocytic evasion (Silva et al., 2017). Viewed through our spatiotemporal-metabolic framework, these failures stem from a core limitation: CSF-1R inhibition targeted the heterogeneous TAM population as a single entity, ignoring the dynamic plasticity and niche-specific adaptation of distinct subsets (Akkari et al., 2020). It did not eliminate all TAMs but instead selected for resistant subpopulations in specific microenvironments—for example, perivascular BAMs (with intrinsic resistance) (Sankowski et al., 2024) and hypoxic core TAMs, which upregulate compensatory survival pathways like IGF-1/IGF-1R (Akkari et al., 2020). This failure was not just due to pathway redundancy, but misdirected targeting: bulk TAM depletion overlooks that metabolically and spatially distinct subsets have unique dependencies and resistance mechanisms. Effective therapy thus demands moving beyond broad depletion to precision target the TAM subsets that predominate in different spatiotemporal contexts.

### 7.2 Novel strategies targeting TAM functional states

Recent advances have shifted focus toward disrupting specific functional circuits that underpin TAM-mediated immunosuppression. Emerging preclinical strategies aim to disrupt specific functional circuits. For instance, studies in mouse models have shown that targeting the creatine shuttle between TAMs and GBM cells (via SLC6A8 inhibition) disrupts metabolic symbiosis in hypoxic niches and enhances T-cell function (Rashidi et al., 2024). However, the translational potential of this approach remains to be evaluated in clinical settings. Similarly, targeting the kynurenine-AHR axis (Section 4.2) reverses T-cell suppression (Takenaka et al., 2019). Promising preclinical data support the use of phagocytosis checkpoint blockade via the CD47-SIRPα axis. In orthotopic mouse models, this approach, particularly when combined with radiotherapy, promotes the phagocytosis of glioma cells and extends survival (Gholamin et al., 2017). Encouragingly, these findings have spurred early-phase clinical trials investigating anti-CD47 antibodies in solid tumors, including GBM (e.g., NCT03990233), although results specific to GBM are still awaited. Another preclinical strategy involves epigenetic reprogramming of TAMs. Research in murine models has shown that BACE1 inhibition suppresses STAT3 signaling in TAMs, thereby reducing IL-10 and TGF-β secretion and promoting a phagocytic phenotype (Zhai et al., 2021). Zman-seq suggests intervening early (e.g., inhibiting TGF-β1 or ANXA1 within 48 h) can block immunosuppressive phenotype acquisition, thereby restoring antitumour immune function. This potential strategy may yield significant synergistic effects when combined with drugs targeting late-stage TAM function, such as CD47-SIRPα blockers or metabolic inhibitors (Kirschenbaum et al., 2024).

Furthermore, niche-specific targeting of border-associated macrophages (BAMs) merits attention for their perivascular localization and pro-angiogenic functions (Sankowski et al., 2024). Unlike conventional anti-angiogenic therapies such as bevacizumab, which broadly target VEGF and often exacerbate hypoxia to drive resistance (Mundt et al., 2022), BAM-directed strategies disrupt their unique niche interactions: blocking the CCL2-CCR2 axis to inhibit BAM recruitment (Zhang et al., 2023; Flores-Toro et al., 2020), suppressing BAM-specific MMP14 to reduce vascular abnormalization (Sankowski et al., 2024; Mundt et al., 2022), interfering with BAM-stromal crosstalk (IL-8/TGF-β with CAFs (Ren et al., 2025), CECR1-mediated with pericytes (Zhu et al., 2017b), and leveraging the BAM surface signature for precision perivascular drug delivery to target pro-angiogenic BAMs.

### 7.3 Combinatorial approaches and clinical translation

The most promising strategies involve rational combinations that simultaneously target TAM adaptability and tumor cell vulnerabilities, with the goal of overcoming resistance mechanisms. Several of these combination approaches are now being tested in clinical trials. Based on strong preclinical rationale, the co-administration of CSF-1R inhibitors with PD-1/PD-L1 blockade was pursued to counter compensatory Treg expansion and enhance T-cell infiltration. Early-phase clinical trials (e.g., NCT02526017) have evaluated this combination, yet the results have underscored the challenges, showing limited objective responses and highlighting the need for better patient stratification (Akkari et al., 2020). Radiotherapy synergizes with CD47 inhibition by inducing calreticulin exposure on tumor cells, enhancing phagocytic clearance (Gholamin et al., 2017). Fatty acid oxidation inhibitors sensitize TAM-rich regions to immunotherapy by reversing lipid-driven immunosuppression (Jiang et al., 2022). As evidenced by the mixed results of the aforementioned clinical trials, the translation of even the most promising preclinical findings remains challenging. Obstacles, such as limited blood-brain barrier penetration of therapeutic agents, on-target/off-tumor effects, and the context-dependent efficacy of immunotherapies in the unique GBM TME continue to hinder progress. Future trials should incorporate spatial biomarker profiling (e.g., HMOX1, LGMN, CD39) to stratify patients and monitor TAM subset dynamics during treatment (Ravi et al., 2022; Rong et al., 2005).

A summary of selected early-phase clinical trials discussed in this section, which target key TAM-related pathways, is provided in Supplementary Table S2.

### 7.4 Future directions and unmet needs

To overcome current limitations, the field must develop more physiologically relevant models that recapitulate TAM heterogeneity and plasticity, such as humanized organoids and spatial omics-integrated murine models (Li et al., 2022). Deciphering the metabolic-epigenetic crosstalk that dictates TAM functional states, such as lactylation and acetylation, will provide novel immunomodulatory targets (Zhang and Jagannath, 2025). Zman-seq data suggest that targeting epigenetic modifiers (e.g., HDACs, BET inhibitors) in combination with metabolic inhibitors may reverse TAM immunosuppression by reshaping their chromatin landscape and functional identity (Kirschenbaum et al., 2024). Engineered nanotherapies and exosomes offer promising avenues for subset-specific TAM targeting with improved blood-brain barrier penetration (Rashidi et al., 2024). Integrating AI-driven multi-omics analyses will be essential to identify novel TAM subsets and therapeutic vulnerabilities across spatial and temporal dimensions (Jain et al., 2023). Translating spatial heterogeneity into clinical practice requires non-invasive biomarkers that can stratify patients by their dominant TAM ecosystem. The relative contribution of BMDMs versus microglia may eventually be inferred from circulating extracellular vesicle cargo carrying subset-specific markers like S100A8/A9 (BMDMs) or TMEM119 (microglia), or from cell-free DNA methylation patterns at lineage-specific loci such as P2RY12 versus CD14 (Pombo Antunes et al., 2021, Miller et al., 2025, Sankowski et al., 2024).

Advanced imaging also holds promise: hyperpolarized ^13^C-MRI can detect lactate flux from glycolytic TAMs in hypoxic niches (McBride et al., 2025), while ^18^F-FMISO-PET may correlate with HMOX1^+^ hypoxic TAM distribution (Leimgruber et al., 2020). TSPO-PET and integrin-targeted tracers could further enable visualization of perivascular and activated myeloid populations (Lanzafame et al., 2026).

Ultimately, clinical trials should incorporate biomarker-based stratification—for example, enrolling ‘BMDM-high’ versus ‘microglia-high’ tumors—with serial monitoring to track dynamic shifts in TAM ecosystems, enabling adaptive therapy strategies aligned with the spatiotemporal framework proposed in this review (Ravi et al., 2022).

In conclusion, targeting TAM heterogeneity in GBM requires a paradigm shift from broad depletion to precision reprogramming of context-specific subsets. By leveraging mechanistic insights from single-cell and spatial biology, future therapies may finally disrupt the immunosuppressive networks that underlie GBM’s therapeutic resistance (Ravi et al., 2022; Pombo Antunes et al., 2021; Rong et al., 2005).

## 8 Discussion

GBM is a primary central nervous system malignancy characterized by a highly heterogeneous immune microenvironment. TAMs, the most abundant immune population within the GBM landscape, play a pivotal role in tumor initiation, progression, and therapeutic resistance (Marusyk et al., 2020; Nicholson and Fine, 2021; Mathur et al., 2024). In recent years, the application of single-cell and spatial omics technologies has fundamentally shifted our perspective from static, simplistic classifications toward a dynamic and contextual understanding of TAMs. This new paradigm captures their continuous functional evolution, metabolic reprogramming, and spatiotemporal dynamics, revealing that TAM identities are not fixed but are dynamically acquired and reshaped by the evolving tumor microenvironment (Lapointe et al., 2018; Stupp et al., 2005; Hoogstrate et al., 2023).

Based on recent studies, we have re-characterized TAMs in terms of their origins (BMDMs, microglia, and BAMs), functional states, metabolic profiles, and interactions with non-neoplastic cells (Friebel et al., 2020; Cui et al., 2020; Strizova et al., 2023). Advances in single-cell sequencing and spatial omics have revealed novel TAM subsets with functional and regional specificity, such as hypoxia-responsive TAMs (hypoxia TAMs) and chemotherapy-responsive TAMs (Chemo-TAMs) (Dusoswa et al., 2020; Chuang et al., 2013; Zhao et al., 2021). Notably, although most TAMs exhibit pro-tumor phenotypes, emerging evidence suggests that certain subsets possess anti-tumor potential under specific conditions. For instance, TAMs reprogrammed by T cell-derived cytokines (e.g., IFN-γ) can downregulate immunosuppressive molecules and synergize with CD8^+^ T cells to enhance tumor cell killing (Trotta et al., 2025). Although these anti-tumor subsets are relatively rare and easily suppressed in the strongly immunosuppressive microenvironment, their existence underscores the extreme complexity of TAM functionality and offers new perspectives for leveraging TAM heterogeneity to augment anti-tumor immunity (Strizova et al., 2023; Trotta et al., 2025).

These functionally distinct TAM subsets exhibit contextual spatial enrichment, as detailed in Sections 3, 6. This function-space coupling highlights the extreme heterogeneity of the TAM population and exposes the limitations of traditional classification frameworks (Strizova et al., 2023). The application of Zman-seq has elucidated a time-resolved differentiation trajectory of monocytes into immunosuppressive TAMs, driven by TGF-β1 and ANXA1 signaling (Kirschenbaum et al., 2024). This discovery adds a new temporal dimension to our understanding of TAM heterogeneity, illustrating that functional states are acquired dynamically rather than being fixed. Targeting this differentiation process during its early phases may represent a promising strategy to counteract TAM-mediated immunosuppression before it becomes entrenched. During progression to recurrence, the TAM compartment remodels fundamentally, shifting from microglia-dominance to a BMDM-enriched pool (Pombo Antunes et al., 2021). This BMDM-enriched ecosystem is a key driver of the heightened therapy resistance and poorer prognosis associated with recurrent GBM (Pombo Antunes et al., 2021, Sun et al., 2024, Zhang et al., 2023). This shift is driven by spatiotemporal dynamics, such as HIF-1α/LGMN axis-mediated PD-L1 upregulation, underscoring their adaptive complexity and contribution to therapy resistance (Pombo Antunes et al., 2021; Pang et al., 2023). Furthermore, TAMs undergo metabolic reprogramming, including enhanced glycolysis and aberrant lipid uptake, synergizing with tumor cells through pathways, such as HEXB-ITGB1/ILK/YAP1, where lactate secretion reinforces IL-10-mediated immunosuppression via histone lactylation (Sankowski et al., 2024; De Leo et al., 2024; Yin et al., 2022). Targeting key metabolic nodes, such as SLC6A8 inhibitors, may disrupt these tumor-promoting pathways (Rashidi et al., 2024). Mitochondrial metabolism also shapes TAM function. Tumor-derived GM-CSF drives OXPHOS and eATP production in TAMs, activating P2X7R on glioma cells to promote proliferation (Wu et al., 2024). Romo1 sustains M2 polarization via mTORC1, while ROS exerts dual effects—moderate levels promote immunosuppression, excessive levels induce glioma cell death (Sun et al., 2020; Guo et al., 2026). These findings reveal additional therapeutic targets within the mitochondrial compartment.

Despite significant advances in understanding TAM heterogeneity, critical gaps remain. The mechanisms by which novel subsets like lipid-enriched LLMs interact with their target cells—particularly glioma stem cells—are still unclear (Yabo et al., 2024; Wei et al., 2020). Communication between distinct TAM subsets and their spatiotemporal evolutionary trajectories throughout glioma progression remains inadequately mapped (Utz et al., 2020; Mundt et al., 2022). The intricate relationship between metabolic and epigenetic states in regulating TAM function across spatial and temporal contexts requires further investigation (Liu et al., 2024; Gabrusiewicz et al., 2016). Large-scale multi-omics approaches hold promise for reconstructing TAM evolutionary trajectories (Mills et al., 2000; Sica and Mantovani, 2012).

Combination strategies show potential against TAM heterogeneity, as exemplified by dual CSF-1R/IGF-1R blockade proven effective in GBM models (Quail et al., 2016), and by JAK/PD-1 inhibition, which has been shown to overcome cytokine-mediated immunosuppression and enhance T cell function in clinical trials for other cancers (Mathew et al., 2024). However, clinical translation remains challenging. For example, CSF-1R inhibitors may fail due to compensatory metabolic adaptations, such as IGF-1 activation (Akkari et al., 2020), while the dynamic remodeling of CD73^+^ TAM populations may undermine the efficacy of immune checkpoint blockade (De Leo et al., 2024; Zhu et al., 2024; Zhang et al., 2018). Moreover, current experimental models (e.g., murine models) inadequately recapitulate the complexity of the human GBM microenvironment, limiting translational reliability (Sattiraju et al., 2023; Kloosterman et al., 2024).

Future breakthroughs may arise from AI-driven multi-omics integration—including spatial transcriptomics, metabolomics, and proteomics—for precise subset identification. Researchers could track TAM dynamics in real time with *in vivo* imaging and tumor organoid models (De Leo et al., 2024; Zhu et al., 2024; Zhang et al., 2018). Metabolic-epigenetic crosstalk—such as hypoxia-induced histone acetylation or lactylation—could yield novel immunomodulatory targets (Palmieri et al., 2017; Zhang et al., 2021; Arlauckas et al., 2018). The application of Zman-seq and similar multi-omic technologies will be crucial for deciphering the epigenetic drivers of TAM plasticity and identifying novel therapeutic targets that disrupt the TAM-tumor metabolic symbiosis (Kirschenbaum et al., 2024). Additionally, engineered exosomes and biomimetic nanoparticles offer promising avenues for enhancing blood-brain barrier penetration and targeted drug delivery (Silva et al., 2017; Takenaka et al., 2019). Multimodal therapies co-targeting TAMs and other microenvironment components represent a promising direction (Wainwright et al., 2011; Goswami et al., 2020; Liu et al., 2019), though challenges remain, including limited spatial resolution, off-target effects, and therapeutic resistance (Quail and Joyce, 2017; Kumar et al., 2016; Lin et al., 2024). Ultimately, a comprehensive multi-omics analysis of GBM-associated TAMs at both macro levels (mapping tumor evolution) and micro levels (uncovering molecular mechanisms) will provide more precise insights and yield promising therapeutic strategies with clinical translational value (Xiong et al., 2022; Sprooten et al., 2024; Fares et al., 2023).

## 9 Conclusion

The spatiotemporal and metabolic heterogeneity of TAMs is a cornerstone of GBM pathogenesis, driving immunosuppression and therapeutic resistance through dynamic interactions within the tumor microenvironment (TME). Our review summarizes multidimensional heterogeneity—spanning TAM origins (BMDMs, microglia, and BAMs), functional plasticity, metabolic reprogramming (glycolysis, amino acid/lipid metabolism), and their dynamic spatiotemporal organization. Single-cell and spatial omics reveal specialized TAM subsets that are preferentially enriched within, rather than strictly confined to, specific niches, a model of context-dependent localization guided by metabolic demands, structural cues, and tumor-derived signals. These subsets engage in dynamic crosstalk and competition, and their composition fundamentally shifts during recurrence towards a BMDM-dominated pool. This spatiotemporal and adaptive complexity is a cornerstone of GBM’s resilience. Critically, TAM composition evolves temporally: microglia dominate primary GBM, while peripheral-derived macrophages expand in recurrence, correlating with aggressive progression. Underpinning this temporal evolution is a complex spatial logic, where TAM localization follows a logic of contextual enrichment guided by metabolic demands, structural cues, and tumor-derived signals, rather than rigid anatomical borders. By elucidating these mechanisms—from metabolic symbiosis to context-dependent spatial distribution—we provide a framework for understanding GBM resilience and highlight promising therapeutic strategies targeting context-specific TAM subsets. Future research should prioritize decoding TAM evolutionary trajectories and microenvironmental interdependencies to inform novel therapeutic paradigms.

## Glossary

2-HG: 2-Hydroxyglutarate
A2A: Adenosine A2A Receptor
ADCC: Antibody-Dependent Cellular Cytotoxicity
ADM: Adrenomedullin
AHR: Aryl Hydrocarbon Receptor
ANXA1: Annexin A1
Ang2: Angiopoietin-2
ApoA1: Apolipoprotein A1
APCs: Antigen-Presenting Cells
Arg1: Arginase 1
ATF4: Activating Transcription Factor 4
ATP: Adenosine Triphosphate
BACE1: Beta-Secretase 1
BAMs: Border-associated macrophages
BCAA: Branched-Chain Amino Acid
BCAT1: Branched-Chain Amino Acid Transaminase 1
BBB: Blood-Brain Barrier
BMDMs: Bone Marrow-Derived Macrophages
BET: Bromodomain and Extra-terminal domain
BNIP3: BCL2 Interacting Protein 3
BTB: Blood-Tumor Barrier
CAFs: Cancer-Associated Fibroblasts
CAR-T: Chimeric Antigen Receptor T-Cell Therapy
CAR-TAMs: Chimeric Antigen Receptor Tumor-Associated Macrophages
CCL2: C-C Motif Chemokine Ligand 2
CCL4: C-C Motif Chemokine Ligand 4
CCR2: C-C Chemokine Receptor 2
CD36: Cluster of Differentiation 36
CD39: Ectonucleoside Triphosphate Diphosphohydrolase 1
CD47: Cluster of Differentiation 47
CD73: Ecto-5′-Nucleotidase
CNS: Central Nervous System
CSF-1: Colony-Stimulating Factor-1
CSF-1R: Colony-Stimulating Factor 1 Receptor
CSTB: Cystatin B
CTLA-4: Cytotoxic T-Lymphocyte-Associated Protein 4
CXCL2: C-X-C Motif Chemokine Ligand 2
CXCR2: C-X-C Motif Chemokine Receptor 2
DCs: Dendritic Cells
ECM: Extracellular Matrix
ECs: Endothelial Cells
EDA: Extra Domain A
EGFRvIII: Epidermal Growth Factor Receptor Variant III
EGFRwt: Epidermal Growth Factor Receptor Wild-Type
EMT: Epithelial-Mesenchymal Transition
EVs: Extracellular Vesicles
FAO: Fatty Acid Oxidation
FAP: Fibroblast Activation Protein
GATM: Glycine Amidinotransferase
GBM: Glioblastoma
GLUT1: Glucose Transporter 1
GPNMB: Glycoprotein Non-Metastatic Melanoma Protein B
HBI: higher cerebral blood flow interface
HDAC: Histone Deacetylase
HEXB: Hexosaminidase B
HIF-1α: Hypoxia-Inducible Factor 1-Alpha
HMOX1: Heme Oxygenase 1
ICIs: Immune Checkpoint Inhibitors
IDH: Isocitrate Dehydrogenase
IFN-γ: Interferon-γ
IGF-1: Insulin-Like Growth Factor 1
IGF-1R: Insulin-Like Growth Factor 1 Receptor
IL-4: Interleukin-4
IL-6: Interleukin-6
IL-8: Interleukin-8
IL-10: Interleukin-10
IL-13: Interleukin-13
ILK: Integrin-Linked Kinase
ITGB1: Integrin Subunit Beta 1
JAK: Janus Kinase
JAK/STAT: Janus Kinase/Signal Transducer and Activator of Transcription
KLF4: Krüppel-Like Factor 4
LAT1: L-Type Amino Acid Transporter 1
LBI: lower cerebral blood flow interface
LDHA: Lactate Dehydrogenase A
LGMN: Legumain
LLMs: Lipid-laden macrophages
LOX: Lysyl Oxidase
MCP-1: Monocyte Chemoattractant Protein-1
MDSCs: Myeloid-Derived Suppressor Cells
M-MDSC: Monocytic Myeloid-Derived Suppressor Cells
MMPs: Matrix Metalloproteinases
OLFML3: Olfactomedin 3
OXPHOS: Oxidative Phosphorylation
PATZ1: POZ/AT Hook/Zinc Finger 1
PD-1: Programmed Cell Death Protein 1
PD-L1: Programmed Death-Ligand 1
PERK: Protein Kinase R-like Endoplasmic Reticulum Kinase
PGK1: Phosphoglycerate Kinase 1
PI3K: Phosphoinositide 3-Kinase
PMN-MDSC: Polymorphonuclear Myeloid-Derived Suppressor Cells
P2RY12: Purinergic Receptor P2Y12
P2X7R: P2X7 Receptor
PRR: Pattern Recognition Receptor
PBZ: Peritumoral Brain Zone
ROS: Reactive Oxygen Species
Romo1: Mitochondrial ROS Modulator 1
S100A8/A9: S100 Calcium Binding Protein A8/A9
SDF-1α: Stromal Cell-Derived Factor-1α
SIRPα: Signal-Regulatory Protein Alpha
SLC6A8: Solute Carrier Family 6 Member 8
SOCS3: Suppressor of Cytokine Signaling 3
STAT1: Signal Transducer and Activator of Transcription 1
STAT3: Signal Transducer and Activator of Transcription 3
TAAs: Tumor-Associated Astrocytes
TAMs: Tumor-Associated Macrophages
TGF-β: Transforming Growth Factor-β
TIE-2: Tyrosine Kinase with Immunoglobulin-like and EGF-like Domains 2
TIM-3: T-Cell Immunoglobulin and Mucin-Domain Containing-3
TLR4: Toll-Like Receptor 4
TME: Tumor Microenvironment
TNF-α: Tumor Necrosis Factor α
TREM2: Triggering Receptor Expressed on Myeloid Cells 2
TMEM119: Transmembrane Protein 119
VCAM-1: Vascular Cell Adhesion Molecule 1
VEGF: Vascular Endothelial Growth Factor
YAP1: Yes-Associated Protein 1
